# The RICORDO approach to semantic interoperability for biomedical data and models: strategy, standards and solutions

**DOI:** 10.1186/1756-0500-4-313

**Published:** 2011-08-30

**Authors:** Bernard de Bono, Robert Hoehndorf, Sarala Wimalaratne, George Gkoutos, Pierre Grenon

**Affiliations:** 1 European Bioinformatics Institute, Wellcome Trust Genome Campus, Cambridge CB10 1SD, UK; 2 Dept. of Genetics, University of Cambridge, Downing Street, Cambridge, CB2 3EH, UK; 3 Auckland Bioengineering Institute, University of Auckland, Symonds Street, Auckland 1010, New Zealand

## Abstract

**Background:**

The practice and research of medicine generates considerable quantities of data and model resources (DMRs). Although in principle biomedical resources are re-usable, in practice few can currently be shared. In particular, the clinical communities in physiology and pharmacology research, as well as medical education, (*i.e*. PPME communities) are facing considerable operational and technical obstacles in sharing data and models.

**Findings:**

We outline the efforts of the PPME communities to achieve automated semantic interoperability for clinical resource documentation in collaboration with the RICORDO project. Current community practices in resource documentation and knowledge management are overviewed. Furthermore, requirements and improvements sought by the PPME communities to current documentation practices are discussed. The RICORDO plan and effort in creating a representational framework and associated open software toolkit for the automated management of PPME metadata resources is also described.

**Conclusions:**

RICORDO is providing the PPME community with tools to effect, share and reason over clinical resource annotations. This work is contributing to the semantic interoperability of DMRs through ontology-based annotation by (i) supporting more effective navigation and re-use of clinical DMRs, as well as (ii) sustaining interoperability operations based on the criterion of biological similarity. Operations facilitated by RICORDO will range from automated dataset matching to model merging and managing complex simulation workflows. In effect, RICORDO is contributing to community standards for resource sharing and interoperability.

## Background

Data and model resources (DMRs) in biomedical research and practice cover a wide range of electronic resource types. In the medical regulatory and clinical domain, for example, drug development trials and patient management practice generate considerable amounts of free-text notes, investigative, analytic and interventional results in tabulated form, various types of image data, mathematical models, as well as associated training and teaching material. The output of basic biological research (*e.g*. drug discovery, tissue biophysics, genomics) is comparably broad and heterogeneous.

The biomedical community is becoming increasingly aware of the importance of DMR standardization, sharing and publication [1]. In turn, a number of funding bodies have established relevant policies in support of a co-ordinated communal DMR sharing strategy (*e.g*. see [2-6]). In particular, the standardization of DMR documentation is fundamental in supporting resource sharing - in principle, the documentation of a resource renders it more accessible to interpretation and consequently encourages its further re-use and interoperability with other resources. In practice, however, the procedure of applying DMR documentation is typically considered to (i) be very time-consuming, and (ii) able to offer only limited support for resource interoperability (*e.g*. see background section of [7]).

In the physiology modeling community, for instance, the documentation and systematic annotation of DMRs is known to face a number of obstacles [8]. For example, due to the relative lack of familiarity with (i) controlled biomedical vocabularies and their key role in DMR annotation, as well as (ii) associated tools that support the automated organization and classification of DMRs, this research community finds little practical incentive to take on the logistic challenge exacted in documenting DMRs over a large scale. A common concern (in discussion with the physiology modelling community by one of us, *BdB personal communication*) about such documentation argues that there is little in the way of communal annotation standards to justify the investment required. In addition, the effort employed by biomedical communities in providing detailed annotation to a DMR tends to be closely influenced by the expectation of a resource being shared [7]. Therefore, the limits imposed on the distribution of a resource (typically for commercial, legal, confidentiality, but also interoperability, reasons) tend to curb directly the quality and machine readability of the corresponding documentation: after all, why document a DMR if the resource cannot (or will not) be accessed by third parties?

The issues outlined above present a formidable obstacle to the communal provision and standardization of DMR documentation in the clinical domain. This paper reports on the ongoing effort to achieve a coherent DMR documentation methodology by three distinct clinical community initiatives in collaboration with the **RICORDO **project [9]. The three community initiatives are:

1. the **Virtual Physiological Human (VPH) Network of Excellence **[10], which aims to apply biomedical research outputs into clinical practice and healthcare industries [11]. In particular, this community fosters the integration of clinical data and models for research purposes in an effort to gain a systemic understanding of pathophysiology and to develop clinical diagnostic tools and medical devices.

2. the **Innovative Medicines Initiative **(**IMI**) [12,13], and in particular the 'Drug & Disease Modeling Resource' (**DDMoRe) **[14] community of modellers in academia and Pharma industry. The aim of the DDMoRe is the creation of a communal infrastructure for model based-drug development by (i) facilitating the continuous integration of available information related to a drug or disease, as well as (ii) supporting the rational management of modelling and simulation workflows.

3. the **mEducator Best Practice Network (mBPN) **[15], that aims to implement and critically evaluate existing standards and reference models in the field of e-learning in order to enable specialized state-of-the-art medical educational content to be discovered, retrieved, shared and re-used across European higher academic institutions.

Communities in the three domains described above - physiology and pharmacology research, as well as medical education (**PPME**) - share the objective of managing heterogeneous clinical DMRs based on their biological meaning. The ability to search and compare datasets, and associated models, based specifically on their **biological knowledge **content would (i) support more effective navigation and re-use of clinical DMRs, as well as (ii) sustain automated interoperability operations based on the criterion of biological similarity and relatedness. Such automated operations include activities ranging from dataset matching, to model merging, and managing complex simulation workflows.

The biological meaning of a resource may be described by its documentation. The management of automated DMR operations in terms of biological meaning, therefore, depends on this documented biological knowledge being explicit and machine readable. In that sense, when a set of clinical DMRs can be consistently related and navigated through explicit meaning in the documentation, such a set may said to be **semantically interoperable**. In addition, when this explicit meaning is machine readable, semantic interoperability operations may be carried out in an automated manner.

In this paper, we outline the efforts of the PPME communities to achieve automated semantic interoperability for clinical DMR documentation in collaboration with the RICORDO project. We first briefly overview current community practices in resource documentation and knowledge management. We then discuss the requirements and improvements sought by the PPME community to the above documentation practices and associated knowledge representation. We then present how the RICORDO community effort addresses the key challenges in creating a representational framework and associated infrastructure for the management of PPME DMRs. In particular, the Results section introduces an ontology-based knowledge representation framework and associated tools that are being developed for the biological annotation and organization of DMR documentation. Furthermore, we show how the RICORDO framework will facilitate the automated management of clinical DMRs based on the biological meaning of resources.

### How do the PPME communities currently manage the biological documentation of DMRs?

**Figure 1:**
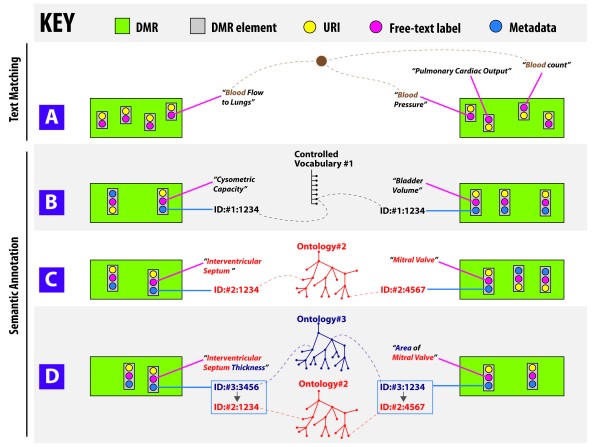
**Comparing biological meaning associated with data and model resource (DMR) elements**. A. Free-text labels associated DMR elements that convey human-readable meaning (*e.g*. text label associated with a data column in a spreadsheet) are a very common method of documentation. Text mining methods can assist with finding relationships between text labels, but may encounter difficulties in identifying closely related concepts expressed using different words: for example the labels "Blood Flow to the Lungs" and "Pulmonary Cardiac Output" have very similar meaning but their textual representation is very divergent. B. Controlled vocabularies provide a standard set of Uniform Resource Identifiers (URIs) with which relevant biomedical concepts may be unambiguously associated. For example, while each of the two elements carries a distinct free-text label, their metadata mappings to the same controlled vocabulary term (with ID#1:1234) makes it explicit that the annotations associated with the two DMR elements are semantically identical (*i.e*. are synonymous). C. Ontologies provide explicit machine readable knowledge about relationships between terms. The above example illustrates the hierarchy of parts of the heart. By explicitly representing knowledge as well-defined concept nodes and relation edges between such concepts, it is possible to compare DMR metadata associated with concepts from the same ontology precisely and automatically. D. Part of the RICORDO effort is to provide tools for the annotation of DMR metadata with composite ontology structures. A composite term consists of two or more ontology terms in which the relationship between such terms is explicitly represented within the composite knowledge structure. Such composites may be compared on the basis of the terms that compose them - for instance, the two composites depicted in this diagram may be compared, using classification tools, on the basis of the ontology terms for cardiac structure (#2: red) and biological qualities (#3: blue) from which they are derived.

In the PPME communities discussed above, clinical DMR documentation is typically carried out at individual project or study level (*e.g*. [16]). In many cases, this documentation is effected by the same project participants who generated the resource in the first place, in the form of **free-text labels **associated with DMR elements [8] (see also Figure 1A). Examples of elements in clinical DMRs include (i) a data column in a clinical trial spreadsheet or database table, (ii) a variable in the code of a physiology model, (iii) a specific spatial region in a radiology image, or (iv) a pathology term in a flat list of disease names.

Free text labels associated with clinical DMRs carry with them a considerable baggage of ***implicit ***biomedical knowledge. Phrases used for free-text labelling vary between different PPME communities and the standardization of such phrases is particularly difficult if the DMRs containing such labels are not shared. In some cases, text mining techniques may assist in relating DMRs based on their label content (see Figure 1A), but such approaches have significant limitations without the use of independent reference knowledge structures [17].

The past decade saw an increased community effort in developing independent reference knowledge structures as a means to standardize the representation of biological meaning in DMRs (*e.g*. [18]), and to render DMR documentation more machine processable and interpretable (*e.g*. [19]). Two key advances in DMR documentation management and semantic interoperability were the development of:

1) *Community semantic metadata standards and associated tools*;

Metadata refers to machine readable documentation material that is linked to a corresponding DMR element indicating how the actual content of that element should be interpreted. Semantic metadata ascribes a DMR element with some meaning. By explicitly representing the meaning of a DMR element, this type of metadata adds semantic features to a resource and provides a machine readable and independent guide as to what a particular DMR element represents. The goal of achieving semantic interoperability for a set of DMRs is motivated by the need to automate the coherent interpretation of DMR content over a large number of diverse DMRs. A key result of attaining this goal is the ability to automatically identify DMRs that are related to each other solely on the basis of their metadata documentation notwithstanding any differences in format, accessibility or ancillary free-text labels the various DMRs may have. The automation of semantic interoperability requires a dedicated computational infrastructure (*e.g*. [20-22]).

**Figure 2:**
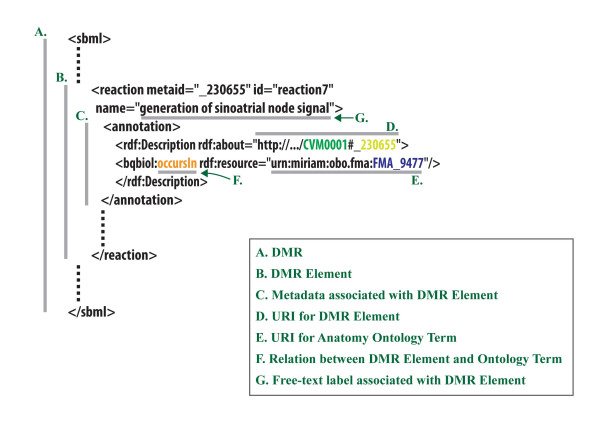
**An example of an annotation triplet in the metadata of a model resource**. Example illustrating the structure of a typical model resource A (in this case, an SBML model) in which element B is shown to bear (i) a human-readable text label G, as well as (ii) machine-readable metadata C. The annotation triplet is composed of a DMR element URI (D), a relation (F), and an ontology URI (E). In the above example, the annotation in the metadata conveys the meaning that the model's reaction with the Unique Resource Identifier (URI) 230655 (D) occurs in (F) the anatomical location identified by URI FMA_9477 (E). The latter URI represents the sinoatrial node term in the Foundational Model of Anatomy [29].

2) *Controlled vocabularies and ontologies (CVOs)*;

CVOs are independent knowledge structures used by the community to provide a standardized set of terms with which to annotate DMR metadata. An example of an annotation using CVOs is shown in Figure 2. In some cases, simple vocabularies are primarily developed to (i) support human readability of metadata and (ii) provide a stable set of Uniform Resource Identifiers (URIs) for annotation. Examples of such terminologies consist of either a flat list (*e.g*. CDISC terminology [23]) or a single hierarchy (*e.g*. MedDRA [24]) of standard terms controlled via some editorial process to avoid semantic redundancy and overlap (hence the use of the phrase 'controlled vocabulary'). Compared to flat-list terminologies, biomedical ontologies aim to render the meaning of their terms explicit and amenable to machine processing and automated reasoning [25]. Ontologies are therefore a more knowledge-rich means by which to standardize the terms used in a domain and to render their meaning explicit. Considerable progress has been made in developing reference ontologies for key domains in biology, including gene functions and processes [26], chemical entities [27], proteins [28], anatomy [29] and phenotypes [30].

Controlled vocabulary flat lists offer some scope for automated processing of knowledge embedded in DMR metadata (see Figure 1B). However, ontologies provide a more detailed representation of relationships between concepts over which DMR metadata may be classified and compared (Figure 1C) [25]. This classification process of automated traversing of, and inference from, this type of knowledge graph is sometimes referred to 'reasoning over an ontology'. This type of automated reasoning is simply not possible with list-based controlled vocabularies.

While the use of CVOs in providing stable identifiers for semantic metadata annotation (exemplified by Figure 1B) has contributed significantly to standardizing DMR documentation methodologies (*e.g*. [18]), this approach is still beset by two key limitations:

1) *Some CVOs may overlap in their knowledge domain without being semantically interoperable*;

Different PPME communities may adopt different CVOs as standard for DMR metadata annotation. However, no explicit mapping between semantically overlapping terms in the distinct CVOs may exist. For example, without an appropriate mapping between MedDRA and CDISC terminologies (*e.g*. via metathesaurii like UMLS [31]), it is difficult to automatically infer that both the MedDRA Lower-Level Term 'Itchy Rash' and the CDISC CodeList Name 'Skin Classification' relate to some property of the skin. If this is the case, then DMR metadata that bears CDISC terms may not be semantically interoperable with DMR metadata using MedDRA terms in an automated manner. This lack of semantic interoperability may present a serious problem with the exploitation of legacy data if heterogeneous standards were applied to DMR documentation metadata.

2) *Technical issues with reasoning over large ontologies*;

Although, in principle, ontologies provide an explicit graph structure over which DMR metadata may be compared, in practice the complexity of large reference ontologies (*e.g*. ontologies for biomedically-relevant small molecules, human anatomy *etc*.) may lead to serious computational performance limitations. These technical limitations often prove to be a formidable obstacle for small isolated PPME communities to benefit from complex knowledge structures. When ontology reasoning is not applied, the role of an ontology in supporting semantic interoperability of resources tends to be reduced to that of a flat-list controlled vocabulary that provides stable IDs for direct metadata comparisons (*i.e*. ontology terms are used for direct ID-to-ID matching shown in Figure 1B rather than for the type of reasoning illustrated in Figure 1C).

### How may the current documentation standards and management of clinical DMRs be improved?

In identifying the above limitations in the utilization of CVOs for DMR metadata annotation, the RICORDO effort was able to compile the following key PPME community requirements to improve metadata management and semantic interoperability of clinical resources:

1) *A communal metadata annotation standard should aim to use CVOs that minimize the chance of knowledge domain overlap*;

A number of terminologies and ontologies have been developed to address some particular representational requirement in biomedicine (see portals at the NCBO [32] and OBO Foundry [18]). Some of these CVOs overlap in the domain of knowledge they represent. The establishment of a DMR annotation standard should aim to minimize such overlap. When such overlap is inevitable, appropriate computational services should map CVOs that are semantically interoperable. In view of the richer knowledge structures ontologies are able to provide, a communal metadata annotation standard should ideally identify relevant biomedical ontologies that are supported and maintained by the community.

2) *CVOs used for DMR annotation should be semantically interoperable;*

Elements in PPME resources often represent very complex concepts (*e.g*. processes in physiology). The development and maintenance of CVOs that cover complex domains is a demanding process that requires significant support and input from the community (*e.g*. see [33-37]). The complexity of this operation may either (i) prevent altogether the construction of an appropriate CVO to cover a particular domain of knowledge, or (ii) lead to the divergent development of overlapping CVOs without provision for automated semantic interoperability between them. In either case, standard methods and relevant tools should be provided to make use of existing ontologies in support of (i) filling gaps in domain knowledge representation and (ii) establish explicit semantic mappings between existing CVO terms respectively.

3) *A communal PPME metadata toolkit is required to effect, share and reason over ontology-based annotations*;

A complementary set of tools is required to support annotation authoring, storage and querying. Authoring tools are required by users in the community to effect annotations on the DMRs they generate - such tools could be web-based for ease of access. In this context, the annotation process requires access to (i) DMR element identifiers, (ii) annotation relationships, as well as to (iii) ontological terms for annotation. It is also envisioned that annotation storage, update and lookup functionalities should be web-based. This imposes hardware requirements on the prospective implementation of an infrastructure to deploy the applications and related data over the web. The query step is required to reason over complex ontologies in order to relate DMR annotations with respect to these independent knowledge structures - this aspect of the infrastructure is therefore required to provide a level of performance that is appropriate for an interactive query.

4) *A common format for DMR annotation needs to be established*;

If an annotation framework is to be applied to heterogeneous resources it is required to support the interoperability of annotations: when brought together, annotations of distinct resources need to be manageable as would annotations of a single resource. Syntactic homogeneity of annotation facilitates the machine readability and uniform interpretation of resource metadata. To this end, for example, the community in the systems biology domain is addressing this goal by introducing a common format for annotating their data and models. The Minimal Information Required In the Annotation of Models (MIRIAM) is a set of guidelines for annotation and curation processes of computational models to facilitate their exchange and reuse [38]. A number of VPH resources are already annotated using MIRIAM, such as SBML [39] and CellML [40]. The Model Format OWL (MFO) is another effort within the systems biology community that is focused on data integration by capturing the SBML structure of biological annotations in OWL-DL to support reasoning, validation, and querying of SBML models [41]. The PPME community should build upon such efforts when establishing communal annotation standards.

5) *A PPME toolkit should support community metadata catalogues*;

PPME resources are encoded over a wide range of formats and are subject to a variety of constraints on their distribution to the rest of the community. A communal PPME annotation framework should ensure the structural integrity and security constraints of clinical DMRs. The provision of metadata catalogues that allow the uncoupling of annotation distribution from that of their corresponding resource is a strategy that has been successfully adopted by clinical communities (*e.g*. [7,20]). In other words, PPME annotations would be accessible as a catalogue for querying by third parties, without having to necessarily provide access to the original models or datasets being catalogued. For example, within a Pharma company, a clinical department may serve a catalogue describing clinical trial data holdings without necessarily providing access to the actual data repositories to unauthorised personnel. Furthermore, the uncoupling of metadata from their corresponding resource has the additional benefit of protecting the integrity of DMRs. No significant change to the format of a DMR may be required if related metadata can be stored in a separate file as long as it holds a mapping to the DMR element URIs. This approach may therefore provide a viable semantic interoperability solution despite the inevitable heterogeneity of resource formats: for instance, cardiac physiology models written in different programming (or markup) languages may share the same metadata standard along with radiological datasets of the heart (which may also be stored over a number of heterogeneous formats).

### The scope of the RICORDO effort

The practice, education, research and industrialization of biomedicine generate large quantities of data, often at great risk or expense. In addition, the study and interpretation of this data typically employs the use of mathematical models based on discrete (*e.g*. statistical) or continuous (*e.g*. infinitesimal calculus) methods. In turn, the validity and robustness of a model, and the results it produces, largely depend on the quality and quantity of data that is applied in its construction and usage. One of the key biomedical research applications of semantic interoperability, therefore, is to help the PPME community find datasets (stored in apposite repositories such as [42]) that are relevant to their modelling and educational goals. Ideally, having found the relevant datasets, the same interoperability framework would be transferable to the workflow that handles data and model interaction. When the same semantic metadata standards are applied across the board, both datasets and models achieve semantic interoperability. Achieving automated semantic interoperability across the board of clinical data and models is the scope of the RICORDO effort.

**Figure 3:**
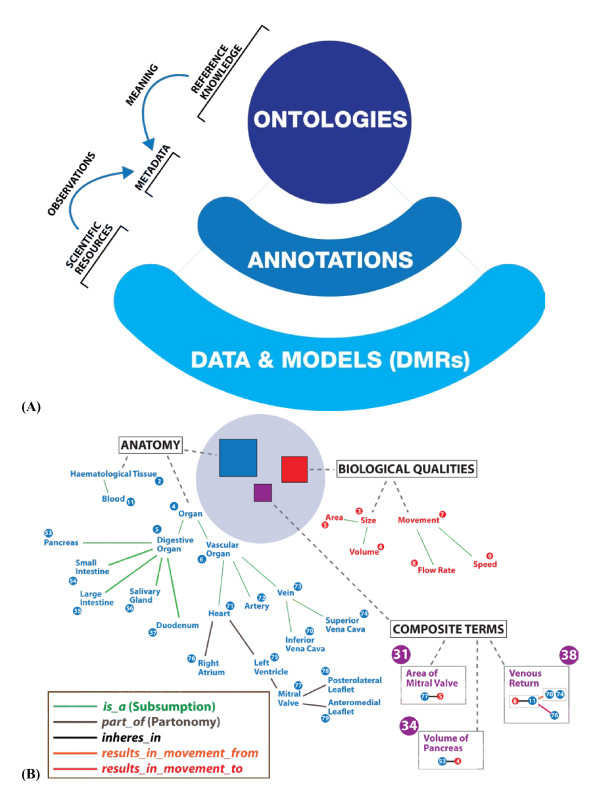
**Ontologies for resource annotation. (A)** Overall schematic representation of the key aspects of semantic interoperability in which annotations provide a link between DMR observations and ontology-based meaning. **(B)** A detail of reference ontology structure representing explicit knowledge. The section of the Biological Qualities ontology only makes use of the subsumption relation. The Anatomy ontology also uses the partonomy relation. Note that, while composite terms have their own unique identifier, they still explicitly refer to Uniform Resource Identifiers (URIs) of standard reference ontologies. In the RICORDO project, both standard reference ontologies and composite terms are formalized in OWL.

The biologically meaningful co-ordination of mathematical modelling and data resource management in the PPME domains requires semantic interoperability between the metadata of clinical models and datasets. To this end, and with reference to the PPME community requirements outlined in the previous section, the RICORDO effort is designing and implementing a semantic interoperability framework over two fronts:

a) The first priority is to contribute to a community standard for:

i. the use of communal and non-overlapping reference ontologies as a source of unambiguous and uniquely identifiable terms and relations for DMR element metadata annotation (Figure 3A);

**Figure 4:**
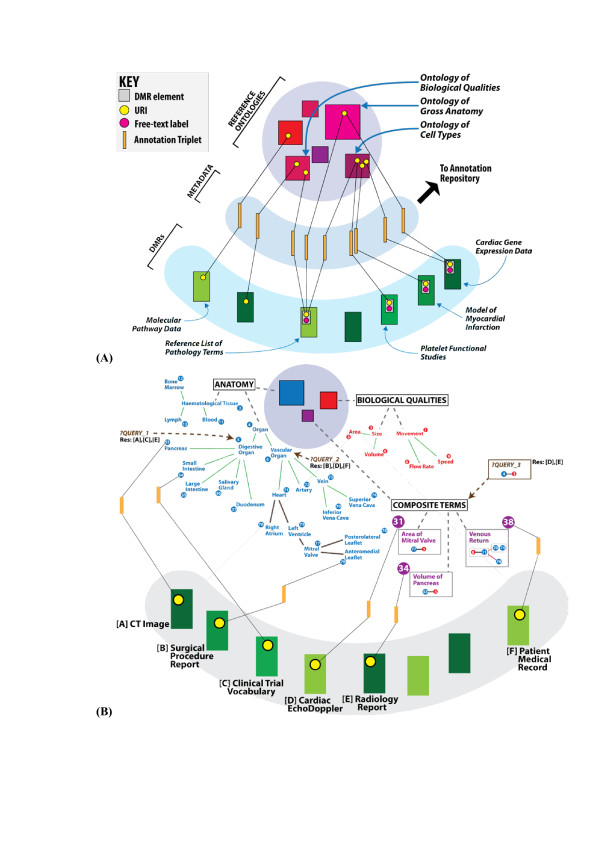
**Annotation metadata. (A)** An example illustrating the role of semantic metadata in support of the semantic interoperability for a set of DMRs. A key requisite is that the DMR metadata must make use of the same set of reference ontologies for its semantic content. Note how, in the case of the 'Reference List of Pathology Terms', a single element URI (yellow dot) is the subject of three distinct annotations to three ontology term URIs. In this particular example, a pathology entity is related to the (i) size quality of (ii) endothelial cells in (iii) the liver - linking the pathology entity URI via distinct relations to URIs respectively from PATO (biological qualities) [45], CellType [67] and FMA (gross anatomy) [29]. In the RICORDO infrastructure the Annotation Repository referred to in this diagram is implemented as an RDF Triple Store. **(B)** The DMR management architecture supports the querying of the repository of annotations using ontology terms as well as composites constructed *ad hoc* for a particular search. **Query_1:** Search for any annotation that involves ontology terms that are a type digestive organ by reasoning over the subsumption relations. This query takes as input the anatomy ontology URI for Digestive Organ (URI: 5). Results: (i) DMR [A] element that represents a pancreatic region on a CT image; (ii) DMR [C] element that bears a clinical trial vocabulary term; and (iii) DMR [E] element on the volume of the pancreas. **Query_2:** Search for annotations to all known vascular organs and their parts. This query takes as input the anatomy ontology URI for Vascular Organ (URI: 6) and returns: (i) DMR [B] element pertaining to the a surgical procedure report about the mitral valve; (ii) DMR [D] element for an cardiac echo Doppler; and (iii) DMR [F] for a medical record about the flow of blood from the patient's central veins to the right atrium. **Query_3:** 'Are there annotations that explicitly describe the size of organs?' The ad-hoc creation of a composite construct that refers to the size that pertains to all known organ subclasses and parts allows the RICORDO framework to ask this question. Results: DMR [D] as well as DMR [E]. Both 'Area' and 'Volume' are subclasses of 'Size' in the quality ontology. 'Pancreas' is a subclass of 'Organ', while 'Mitral Valve' is a part of the 'Organ' subclass 'Heart'.

ii. the well defined representation and encoding of uniquely traceable metadata in which annotations are embedded.

b) The second priority addresses the development of an open toolkit to:

i. support the representation of complex biomedical concepts using terms from standard reference ontologies (known as ontology composites), thus supporting community efforts to fill gaps in the knowledge domain (such as physiology and pharmacology - see 'Key issues' section below) and to improve the semantic interoperability of existing CVOs (Figure 3B);

ii. annotate DMR metadata and to enable the sharing of annotation triplets that are generated by this process (Figure 4A). The distribution of annotations may be uncoupled from the accessibility or format restrictions that may be applicable to their corresponding DMRs;

provide services in support of querying repositories of annotations through efficient automated reasoning over the standard reference ontologies (and their composites) from which annotation terms are derived (Figure 4B).

### Key issues for complex knowledge biomedical representations in physiology and pharmacology

As a field of research, 'physiology' studies the physical principles that govern the behaviour of anatomical structures within processes of medical relevance. This effort overlaps considerably with that of 'pharmacology' and 'systems biology'. As a domain of knowledge that functionally bridges anatomy-level structures to processes (typically through the application of physics), the physiology domain is also sought to provide a clinical knowledge framework that links anatomical abnormalities with pathological processes.

Clinical terms from pharmacology and physiology are employed by the biomedical community to annotate DMRs that are relevant to drug development and clinical practice respectively. A significant proportion of such terms (e.g. 'cardiac output', 'blood pressure') refer to canonical notions of biological structure (*e.g*. anatomy, molecular architecture) and process (*e.g*. drug action, physiological mechanisms), whilst others refer to pathological deviations from anatomical (*e.g*. aortic aneurysm) and processual (e.g. respiratory failure) norms [43].

Clinical terms carry significant implicit clinical knowledge and cannot easily be interpreted by non-experts or machines. For instance, the close biological similarity of the terms 'cardiac angina' and 'intermittent claudication' - both involve pain due to the process of ischaemia that is usually the result of underlying atherosclerosis - may not be immediately obvious. Similarly, it may be difficult for a non-expert to interpret and relate terms such as 'renal clearance', 'cystometric capacity' and 'venous return'. In this particular example, 'cystometric capacity' and 'venous return' both represent the notion of volume of some biological structure (urinary bladder in the former and blood in the latter), 'renal clearance' and 'venous return' both refer to first derivatives with respect to time, while 'renal clearance' and 'cystometric capacity' both describe some functional aspect of the urinary tract.

The above examples show that clinical terms may be implicitly related to one-another in a number of ways. A key step in rendering the knowledge represented by clinical terms explicit is to map the terms to a formal knowledge representation language that enables the description of canonical notions of biological structure and process. Ontologies provide an explicit representation of biological knowledge and biological concepts through axioms and definitions [44]. By mapping clinical terms to reference concepts in ontologies, it is possible to search, relate and classify such terms on the basis of the explicit and formal features described in the ontologies (see Figure 1D for an example).

While considerable progress has been made in developing reference ontologies for key domains in biology, so far, no significant reference ontology or terminology for the domain of physiology has been developed. The key challenges for developing a physiology ontology are in the diversity of the knowledge required to formulate key physiological representations. In addition, the domain of physiology is complex and multi-dimensional, combining domains from the molecular to the organismal level of granularity. Furthermore, physiological phenomena require a complex conceptualization.

By mapping clinical terms onto biological concepts in ontologies, it is possible to search, relate and classify such terms on the basis of the independent context the ontology graphs provide (see Figures 1D and 4B). A more unified and explicit representation of clinical terms, and by extension disease terms, may therefore be achieved if they were also mapped to standard reference ontologies built by experts in physics, biological processes and structural biology. The RICORDO project aims to use standard reference ontologies maintained by the OBO community [18] as the source of concepts with which to describe complex clinical phenomena. This approach sets the stage for the physiology and pharmacology community to benefit from some of the successes already achieved by the molecular and systems biology community in the biological integration of their DMRs through the use of ontologies (*e.g*. [18,26,45]).

## Results

RICORDO makes use of an interoperability strategy, based on the use of standard reference ontologies, initiated by the molecular [26] and systems biology [46] communities. In the RICORDO framework, terms from a core set of biomedical reference ontologies [18] that convey biological meaning are embedded in DMR metadata.

For ontology-based interoperability solutions for DMRs to be adopted by industrial and clinical communities, significant progress needs to be achieved, and demonstrated in practice, in effecting, sharing and reasoning over annotations. To this end, RICORDO is developing a toolkit that supports community annotation and interoperability requirements discussed previously (see also published project reports [47-50]). In this section, we discuss the results achieved so far in developing the RICORDO framework.

### (a) Ontologies for annotation

#### (a.1) Ontology standards

The goal of achieving semantic interoperability for DMRs in specific domains of biological knowledge leads to the following question: which biological ontologies should be used for DMR annotation? Ideally, the selected ontologies should be (i) well established, (ii) actively supported by the community, and (iii) already being applied in the annotation of biomedical resources in the public domain. Such ontologies would therefore provide the meaning with which to manage considerable biomedical resources already available in the public domain. Furthermore, ontologies that are held as reference standard by the community are more likely to add substantial knowledge to DMRs that are annotated using their terms.

To this end, the initial RICORDO effort has identified a first set of reference ontologies that represent biological structure across multiple scales, starting from small molecules (*e.g*. glucose from ChEBI[51]) and reaching gross anatomical level (*e.g*. spleen from the FMA[29] - see published report [52] for further details). These ontologies have minimal overlap between each other, and their development and maintenance is driven by the community (following OBO principles [18]). A second set of ontologies has been selected to cover biological qualities observed in the lab or clinic (*e.g*. pressure, mass, concentration *etc*.), biological processes, as well as units of measurement [51].

#### (a.2) A grammar to build composite complexes from basic ontology terms

While well-developed reference ontologies are readily available to describe basic biological concepts (e.g. structure, processes and their qualities) in a consistent manner, most biomedical data and models tend to represent more complex concepts as well. An example of a complex concept from physiology is 'venous return', which refers to the rate of blood flowing from the central systemic veins back to the right atrium of the heart. In such a case, no single ontology from the above reference sets can provide a term that completely and explicitly represents the precise meaning of that semantic entity.

In this context, the relevant questions that RICORDO is addressing are: "(i) Could terms from basic reference ontologies be combined into a composite structure that conveys such a complex meaning? (ii) Could such a composite term still be used for annotation and query purposes?"

To address these questions, RICORDO is developing a grammar (and is implementing a corresponding composite term editor - see Toolkit section below) that draws upon terms from basic reference ontologies to create composite representations of complex biological concepts (see Figure 3B for an illustration of the grammar as applied to "venous return", as well as [53]). The key advantage of the composite approach is that complex concepts retain a mapping to reference ontology terms in a systematic and consistent manner (see also published report [54]).

### (b) Metadata standards for annotation with ontology terms

The process of annotation renders knowledge about DMR elements more explicit. For the purpose of semantic interoperability in RICORDO, this annotation is carried out using standard reference ontology terms or their composite constructs (as described above).

The manner by which annotations are embedded in DMR semantic metadata is a crucial aspect of the annotation process. The metadata standard specifies the precise syntax and semantics that relate a DMR element to the terms or composite constructs that are chosen to represent its meaning. This standard is also critical in the development of protocols (and, therefore, tools) that effect and parse DMR annotation metadata. In addition, metadata standards for DMRs carry considerable implications as to how annotations may be stored and shared (i) within the confines of a single organization or, indeed, (ii) with the rest of the community in the public domain.

In RICORDO, annotation-bearing metadata is encoded using the Resource Description Framework (RDF), which has a serialisation in the Extensible Markup Language (XML). RDF is adopted to provide traceable links to triplets of DMR element and ontology concept URIs. These triplets are then collected into an apposite RDF repository and queried using the RDF query language (SPARQL [55]). This strategy can be combined with existing annotation standards such as MIRIAM [38]. RICORDO is implementing an annotation tool that generates such RDF statements - see Toolkit section below.

### (c) Automated reasoning and inference over annotations

It is essential that the expense and commitment invested by an organization to adopt community-wide ontology and metadata standards for annotation is amply matched by the returns of improved DMR interoperability and searchability. Consequently, the contribution of reference ontologies to interoperability ideally should:

(i) exceed the mere provision of an identifier namespace, and

(ii) contribute to the inference of semantic similarity of DMR elements in a manner that is based on much more than the simple matching of identical annotations.

A more productive semantic interoperability approach takes full advantage of the knowledge captured by the (i) reference ontologies, and (ii) DMR annotations, on the basis of well-defined ontological relationships. The use of OWL-based reasoning tools (such as Pellet [56]) in such approaches would carry out logical operations over the graph structure of ontologies in support of the automated classification of DMR annotations (see published report on the RICORDO prototype we have developed that makes use of such reasoning tools [50]).

**Figure 5:**
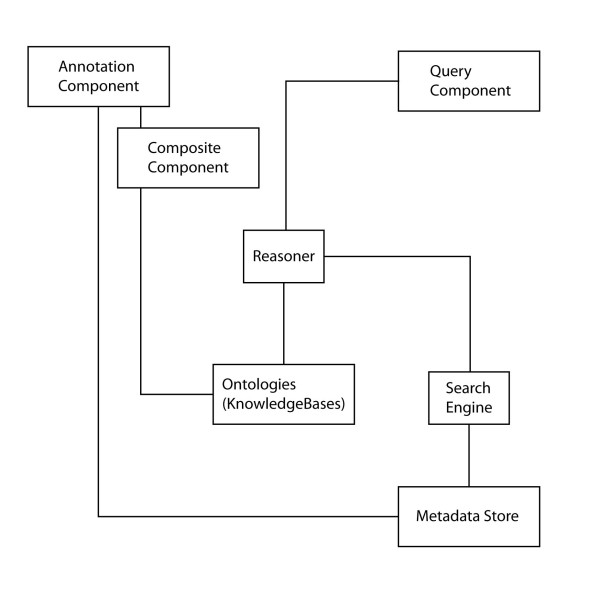
**Schematic overview of the RICORDO ToolKit architecture**. The RICORDO Toolkit is modular and consists of several components. These components can be combined to implement the workflow we envision for annotation of DMRs. The Composite Component enables the creation of composite terms and accesses and modifies the ontologies used by the RICORDO ToolKit. The Annotation Component creates annotations of DMRs and deposits them in the Metadata Store. The Query Component combines reasoning over ontologies and access to the Metadata Store to perform powerful and expressive queries over DMRs. The Composite Component, Annotation Component, Query Component and the Metadata Store are accessible to outside users either directly or through the prototypical RICORDO demo application [64] which integrates and combines the RICORDO Toolkit's components.

To this end, a key requirement of the OWL-based RICORDO reasoning module is to provide efficient performance in its inferences over ontologies of substantial combined size and complexity such as the FMA and ChEBI. The reasoning module we have developed is closely linked to the RDF store that houses annotation triplets (see Figure 5), and the role of the reasoner is to generate the list of relevant ontology terms with which to search the RDF triple store of annotations (*e.g*. to generate all cardiac parts that are known in the anatomy ontology, in order to search the RDF store for all these parts). Examples of reasoning-based queries are outlined in Figure 4B, and the ToolKit section that follows refers to online demo and tutorial materials that illustrate the functionality of this reasoning module.

### (d) The RICORDO ToolKit

The overall strategy of the RICORDO effort is to develop and demonstrate the effectiveness of tools for the academic and industry communities to support interoperability of DMRs using ontologies. To that end, RICORDO is developing a framework of tools to address the requirements we have identified. In particular, we are developing a comprehensive toolkit that facilitates (i) the creation of composite terms, (ii) the annotation of DMR metadata using either composite terms or individual terms from selected reference biomedical ontologies, (iii) the semantic integration of DMRs, and (iv) the retrieval of DMRs based on complex queries over biomedical ontologies. Figure 5 presents schematically the ToolKit framework that (i) enables the creation of composite terms from reference ontologies, (ii) annotates resource metadata and (iii) makes use of automated reasoning over ontologies.

The RICORDO toolkit we are developing consists of four core components:

1. The RICORDO Composite Component enables the creation of composite terms based on the RICORDO core ontologies. This component ensures that the composite terms conform to the RICORDO grammar. To make this complex grammar accessible to users, we have identified and implemented several commonly occurring definition patterns that serve as templates for term creation.

2. The RICORDO Annotation Component enables the creation of annotations of DMRs. In particular, it creates the link between a composite term, or a term in a reference ontology, and a DMR element. If an annotation with a composite term is required, and such a composite term does not exist already in the knowledgebase, the RICORDO Composite Component is used to create this complex term and subsequently generate the annotation.

3. The RICORDO Metadata Store allows the storing and integration of DMR metadata. It contains the annotation triplets and makes them accessible via a standard interface.

4. The RICORDO Query Component is the central component for the retrieval of DMR metadata based on the complex class descriptions contained in the RICORDO core ontologies. The Query Tool makes extensive use of automated reasoning over ontologies and therefore enables complex and precise queries over DMRs. We have implemented patterns to query DMR metadata based on commonly used class definition patterns. The performance level achieved enables real-time response to queries.

These components address some of the major aspects of the RICORDO plan for interoperability of resources in physiology as follows:

1) complex physiological phenomena can be described using the Composite Component,

2) the above composite descriptions, or terms from reference ontologies, can be attached to DMR elements using the Annotation Component,

3) the Metadata store will integrate these annotations across different resources, domains and communities, and

4) the Query Component will allow retrieval of these annotations while combining knowledge from the annotations and the biomedical ontologies developed across communities.

For example, to annotate an element that represents the "Volume of Pancreas" in a radiology resource (see Figure 4B), the Composite Component of the RICORDO Toolkit is first used to create a formal description of "Volume of Pancreas" by combining information from three biomedical reference ontologies. Specifically, "Volume of Pancreas" combines the term "Volume" from the PATO ontology of qualities, the relationship "inheres_in" from the OBO Relationship Ontology [57], and the anatomical term "Pancreas" from the FMA. Second, the Annotation Component is used to link the resource element and the corresponding composite term in a triplet consisting of an identifier for the resource element (in Figure 4B, this element is depicted to originate in a Radiology Report), a relation and a reference to the composite term (in Figure 4B, this composite term is identified by the number '34'). The link created by the Annotation Component is subsequently deposited in the Metadata Store. Using the Query Component of the RICORDO Toolkit (Figures 4B and 5), this annotation can be retrieved using complex queries over both the composite terms and reference ontologies. For example, it is possible to retrieve the annotation with the composite "Volume of Pancreas" by querying for "Size" that inheres in "Organs" (Figure 4B, Query_3).

To support software developers in (i) implementing the standards and (ii) re-use the tool source code we are developing, we make the RICORDO Toolkit prototype freely available on our website[58], under the Apache License 2.0. In addition, we have developed demonstration software that implements all components of the RICORDO Toolkit and enables users to explore the RICORDO functionality (accessible through [59]). We also make a detailed tutorial for using the RICORDO Toolkit available in our website (see [59], documentation section]. Further available resources on the same webpage include project documentation reports (known as Deliverable Reports) as well as links to community efforts that use RICORDO methods and standards (also refer to the Use Cases section that follows).

### (e) RICORDO Use Cases

The RICORDO approach is already being applied to the annotation of resources in three distinct areas, namely the annotation of:

1) biomedical imaging ranging from (i) images (*e.g*. radiology data in DICOM format [60]) to (ii) spatial models (*e.g*. FieldML computational models [61], geometric radiology models[62] and 3D gene expression atlases[63,64]);

2) predicted properties of molecular entities, in particular the output of machine-learning tools predicting protein sequence subcellular localisation [65];

3) variables encoded in physiology models based on ordinary differential equations (ODEs) (*e.g*. [8,39,66]) that represent biophysical measurements relevant to human biology.

## Discussion and Conclusion

The RICORDO effort is based on formal knowledge representation methods, including the use of ontologies, and associated tools. This approach uses the explicit representation of anatomical and medical knowledge in the management of DMR annotation. These annotations, which constitute the resource metadata, are statements mapping ontology term identifiers onto resource element identifiers. Ontologies facilitate machine processing, standardisation of resource metadata, as well as reasoning. The resulting method allows the navigation and querying of annotation repositories using formalized biomedical knowledge. A consequence of this approach is that the process of DMR documentation in the PPME domains is more efficient and has a beneficial impact on resource sharing, as well as fostering the development of communal documentation standards.

RICORDO primarily aims to support the management of heterogeneous biomedical DMRs. The RICORDO framework will bring resources together through a common process of annotation. As a result, these resources will form an ecosystem that can be navigated on the basis of communal reference knowledge and meaning - this is the operational definition of 'resource semantic interoperability' in RICORDO.

The knowledge management workflow we are developing consists of three key steps. The first entails the creation of PPME resource annotation that is machine processable and uses reference and standardised ontology terms. This is followed by the storage of annotations in repositories that are distinct and independent from those containing the original resources. The final stage allows the querying of annotations to retrieve references to relevant resources. This step is enhanced by intermediate domain ontological reasoning.

In this paper, we presented the RICORDO approach applied to the management of clinical data and models and outlined some of the advantages of managing clinical resources with ontologies. The benefits of this approach include the provision of:

1) unambiguous resource annotations;

2) machine processable annotations;

3) inferencing on annotations;

4) the use of biological knowledge in reasoning.

The above contribute directly to the overall goal of RICORDO in supporting semantic interoperability of biomedical DMRs through ontology-based annotation. Achieving such a goal would (i) encourage more effective navigation and re-use of clinical DMRs, as well as (ii) sustain interoperability operations based on the criterion of biological similarity. Such operations include activities ranging from automated dataset matching to model merging and managing complex simulation workflows. This aim is pursued through the:

1) standardisation of metadata, as well as of a core set of reference ontologies for use in annotations;

2) provision of tools to extend and combine ontologies, and query annotations.

RICORDO therefore offers a number of potential advantages to clinical data management by:

1) performing and maintaining annotation of resources while respecting their integrity and confidentiality constraints;

2) bridging clinical terminologies to ontology-based semantics;

3) supporting semantic integration in the physiology and clinical domains and, by extension, the semantic interoperability of their DMRs.

The ongoing RICORDO effort is working closely with knowledge representation and modelling communities to support the development and adoption of semantic interoperability standards and technologies for biomedical research. While the interoperability solutions emerging from RICORDO are principally focused on multiscale biological structure, processes and associated qualities, the application of these solutions may be extended to any domain that is supported by well-established standard reference ontologies.

In addition, RICORDO will provide a metadata management system that extracts and serves annotations via a separate repository service that does not require the public availability of the DMR to which these annotations were originally applied. In practice, therefore, this system will allow users to make well-defined details of their work known to the community, while satisfying the constraints and obligations of confidentiality that sensitive clinical or commercial work often entails. In that sense, the RICORDO approach will make it easier for the community to be aware of the presence of datasets or models that may be relevant to some biomedical objective, despite the fact that the actual DMRs themselves may not be publicly available.

The next challenge for the RICORDO effort is to work with both ontology and modelling communities to establish appropriate training resources in support of the adoption of semantic technologies. This step ensures that users considering the adoption of the RICORDO framework are able to match precisely their DMR interoperability requirements to the rewards and limitations of available semantic solutions.

## Competing interests

The authors declare that they have no competing interests.

## Authors' contributions

BdB, RH, SW, GG and PG carried out the research discussed in this paper. BdB wrote the main text and supplied all figures. RH, SW, GG, PG provided text contributions. All authors read and approved the final version of the manuscript.
